# Pharmacogenetic Association between *XRCC1* Polymorphisms and Response to Platinum-Based Chemotherapy in Asian Patients with NSCLC: A Meta-Analysis

**DOI:** 10.1155/2020/3520764

**Published:** 2020-10-22

**Authors:** Ningning Zhang, Yushu Ouyang, Jianlan Chang, Ping Liu, Xiangyang Tian, Junyan Yu

**Affiliations:** ^1^Department of Oncology, Peace Hospital of Changzhi Medical College, Changzhi, Shanxi 046000, China; ^2^Department of Intervention, Guangdong Provincial Hospital of Chinese Medicine, The Second Clinical Medical College of Guangzhou University of Chinese Medicine, Guangzhou, Guangdong 510005, China

## Abstract

**Background:**

Platinum-based chemotherapy plays an antitumor role by damaging DNA. X-ray repair crosscomplementing protein 1 (XRCC1) participates in DNA repair and thus affects the sensitivity to platinum drugs. Two polymorphisms of *XRCC1*, rs25487 (Arg399Gln) and rs1799782 (Arg194Trp), have been widely studied for the association with clinical outcomes of platinum-based chemotherapy in Asian patients with non-small-cell lung cancer (NSCLC), but the results remain inconclusive. Thus, we performed the present meta-analysis.

**Methods:**

Literature search was performed in PubMed, Web of Science, and EMBASE up to June 2019. Odds ratios (ORs) for objective response ratio (ORR), Cox proportional hazard ratios (HRs) of overall survival (OS) and progression-free survival (PFS), and the corresponding 95% confidence intervals (95% CIs) were calculated to assess the association strengths between *XRCC1* polymorphisms and clinical outcomes. Comparisons were performed in homozygous, heterozygous, dominant, and recessive models.

**Results:**

Finally, a total of 23 studies involving 5567 patients were included in the meta-analysis. Compared to ArgArg of rs25487, GlnGln (OR = 1.71, 95% CI: 1.16-2.52, *p* = .007, *I*^2^ = 56.8%) and GlnArg (OR = 1.23, 95% CI: 1.07-1.40, *p* = .003, *I*^2^ = 29.0%) were associated with higher ORR. Meanwhile, GlnGln indicated a favorable OS (HR = 0.60, 95% CI: 0.40-0.88) and PFS (HR = 0.64, 95% CI: 0.46-0.90). We also found positive associations between rs1799782 and ORR in all comparison models with low between-study heterogeneity. The association strength increased with the number of variant alleles (TrpTrp vs. ArgArg: OR = 1.73, 95% CI:1.31-2.27; TrpArg vs. ArgArg: OR = 1.28, 95% CI: 1.06-1.55), suggesting a gene dosage effect. In addition, TrpTrp predicted a longer OS.

**Conclusion:**

Our results showed that rs25487 and rs1799782 of *XRCC1* are potential markers to predict clinical outcomes of platinum-based chemotherapy in Asian patients with NSCLC.

## 1. Introduction

Lung cancer is the most prevalence malignant tumor and the leading cause of cancer deaths worldwide. It accounts for about 13% of newly diagnosed cancers and is responsible for 17.6% of cancer-related deaths each year^1^. Lung cancer can be histologically classified as non-small-cell lung cancer (NSCLC) and small cell lung cancer (SCLC). NSCLC, accounting for ~85% of all cases, includes several subtypes: squamous cell carcinoma, adenocarcinoma, large cell carcinoma, and the other subtypes^2^. The prognosis of lung cancer is usually poor since only a few patients can be early diagnosed and surgically treated with a good prognosis^3^. Approximately 70% of lung cancer patients develop to advanced stage upon diagnosis^4^.

Platinum-based chemotherapy has been the standard treatment of advanced NSCLC for decades^5^. This chemotherapy is usually in combination with gemcitabine, vinorelbine, paclitaxel, pemetrexed, and so on, yielding a response rate of 30%, a median overall survival of 12 months, and a median disease-free survival of 6 months^6^. Although molecule-targeted drugs have been recommended as the first choice for advanced patients with driver gene mutations in recent years, platinum-based chemotherapy is still the first-line therapy option for those with wildtype genes^7^. However, there is great variability in terms of response to platinum-based chemotherapy among NSCLC patients, even among those at the same stage. Knowing the prognostic factors can help schedule individualized treatment and improve the clinical outcomes of platinum-based chemotherapy.

Platinum drugs, including cisplatin and carboplatin, exert the antitumor effect by binding to DNA, forming DNA adducts that lead to intrastrand or interstrand crosslink and finally inducing severe dislocation of DNA double helix^8^^,^^9^. Treatment failure is mainly caused by resistance to platinum agents. Therefore, the DNA repair pathway, which detects and repairs these damaged DNA, plays a pivotal role in modifying the treatment efficacy of platinum-based chemotherapy in NSCLC.

There are four mainly different DNA repair mechanisms: nucleotide excision repair (NER), base excision repair (BER), mismatch repair (MMR), and double-strain break repair (DSB)^10^. X-ray repair crosscomplementing protein 1 (XRCC1), belonging to the BER pathway, interacts with DNA polymerase-beta, DNA ligase III, and PARP (poly ADP-ribose polymerase) to repair damaged DNA, including platinum-induced damage^11^^,^^12^. Thus, increasing the DNA repair capacity of XRCC1 decreases the clinical response^13^.

There are two polymorphisms of *XRCC1*, rs25487 (Arg399Gln) and rs1799782 (Arg194Trp), that are believed to change the DNA repair activity and affect the sensitivity of tumor cells to platinum drugs^14^. Thus, they have been widely investigated for the association with response to platinum-based chemotherapy in NSCLC patients^15^^–^17, mostly in Asian populations^18^^–^22. However, the results remain inconclusive. In the present study, we aim to evaluate the association between these two polymorphisms in *XRCC1* and clinical outcomes of NSCLC treated with platinum-based chemotherapy in Asian populations.

## 2. Materials and Methods

### 2.1. Literature Search

This meta-analysis was in accordance with the PRISMA checklist. We performed literature search on electronic databases including PubMed, Web of Science, and EMBASE using the following keywords and their combinations: (“X-ray repair cross complementing group 1” or “*XRCC1*” or “rs25487” or “rs1799782”) AND (“platinum” or “cisplatin” or “carboplatin”) AND (“lung cancer” or “NSCLC”) prior to June 2019. Studies investigating the association between *XRCC1* polymorphisms and chemotherapy efficacy of NSCLC were retrieved. The language was restricted to English. Additional articles from the reference lists of reviews and retrieved articles were manually searched to avoid missing eligible studies.

### 2.2. Inclusion and Exclusion Criteria

Eligible studies should fulfil the following criteria: all patients were confirmed as NSCLC (1); all patients received platinum-based chemotherapy (2); all patients were Asians (3); genotype data of rs25487 or rs1799782 were provided (4); and objective response rate (ORR), overall survival (OS), or progression-free survival (PFS) was reported as the outcomes of efficacy assessment, and adequate data were given for the present meat-analysis (5). Studies involving small-cell lung cancer (SCLC) patients or duplicated with other studies or performed in animals or cell lines were excluded. Reviews and case reports were discarded.

### 2.3. Data Extraction

The following information for each eligible study was extracted: first author, year of publication, country, tumor stage, chemotherapy regimens, genotyping method, sample size (male and female), age of participants, genotype distribution, and clinical outcomes. Literature search, filtering, and data extraction were performed by two independent researchers (NZ and YO), and any discrepancy was solved by further discussion with a third researcher.

### 2.4. Quality Assessment

We assessed the quality of all eligible studies by using the Newcastle-Ottawa Scale (NOS, http://www.ohri.ca/programs/clinical_epidemiology/oxford.asp). NOS contains 8 items from 3 domains (selection, comparability and outcome) and has a total of 9 stars. Studies with 6 or more stars were considered of high quality.

### 2.5. Definition of Outcomes

ORR was classified into four categories according to RECIST (Response Evaluation Criteria in Solid Tumors)^23^: complete response (CR), partial response (PR), stable disease (SD), and progressive disease (PD). The responding group included CR and PR while the nonresponding group included SD and PD. OS was defined as the time from starting treatment to the death from any cause or the last follow-up. PFS was defined as the period from the date of treatment to disease progression or death from any cause.

### 2.6. Statistics

Odds ratio (OR) and its 95% confidence interval (95% CI) were calculated to assess the strength of association between *XRCC1* polymorphisms and ORR of NSCLC receiving platinum-based chemotherapy. Hazard ratio (HR) with corresponding 95% CI was calculated to assess the association strength of polymorphisms with OS or PFS. *I*^2^ test and *Q* test were performed for the assessment of between-study heterogeneity. If no obvious heterogeneity existed (*I*^2^ < 50% and *Q* test, *p* > 0.1), a fixed-effect model was applied; otherwise, a random-effect model was used. Meta-regression regarding year of publication, sample size, median age of participants, percent of male, smoking rate, percent of stage IV patients and histological types, and sensitivity analysis were performed to identify the potential source of heterogeneity. Publication bias was assessed by funnel plots and Egger's test. All of the above analyses were performed in the following genetic models of rs25487 or rs1799782: homozygous model (homozygous variant vs. wildtype), heterozygous model (heterozygous variant vs. wildtype), dominant model (homozygous+heterozygous variant vs. wildtype), and recessive model (homozygous variant vs. heterozygous variant+wildtype). STATA v11.0 (STATA Corporation, College Station, TX, USA) was used for meta-analysis, and a *p* value<.05 was considered statistically significant.

## 3. Results

### 3.1. Basic Characteristics

**Figure 1:**
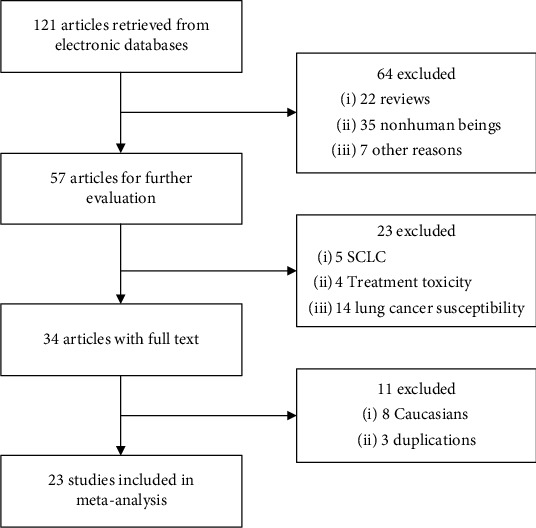
Flowchart of literature search of the present meta-analysis.

A total of 121 potentially relevant articles were retrieved from literature search, and finally, 23 studies^18^^–^22^,^^24^^–^41 comprised of 5567 NSCLC patients were eligible and included in our analysis (Figure 1). Among them, all studies investigated rs25487 and 11 investigated rs1799782 on their association with chemotherapy efficacy. Twenty-one studies only recruited advanced NSCLC patients while 2 included patients with all stages^29^^,^^33^. ORR, OS and PFS were reported in 21, 14, and 5 studies, respectively. Thirteen and ten studies were awarded 6 and 7 stars, respectively, according to NOS, and they were considered of high quality. The basic characteristics of all included studies were listed in Table 1.

### 3.2. Rs25487

#### 3.2.1. Objective Response Rate

**Figure 2:**
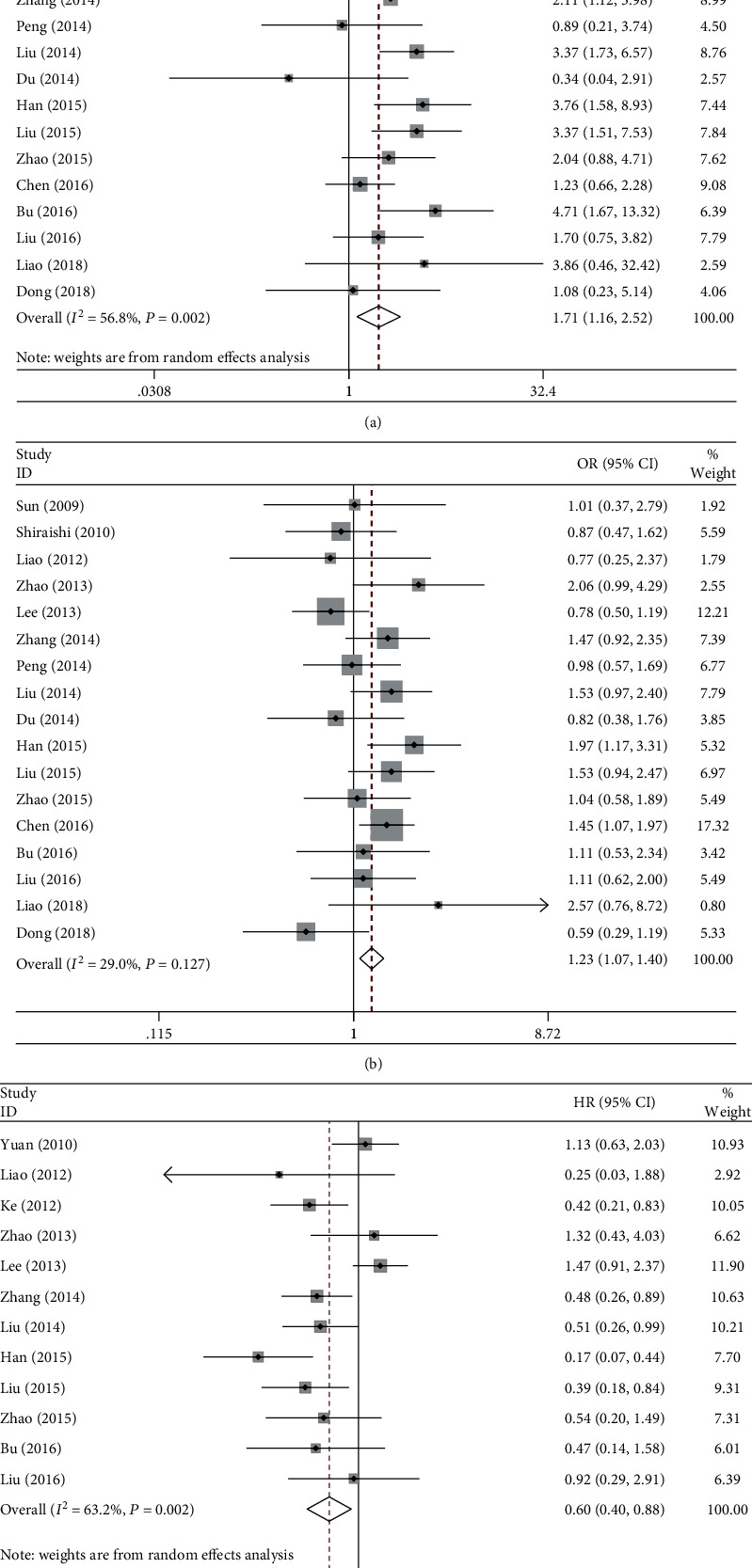
Forest plots of rs25487 (Arg399Gln) and clinical outcomes in NSCLC patients under platinum-based chemotherapy. (a) ORR in the homozygous model. (b) ORR in the heterozygous model. (c) OS in the homozygous model. NSCLC: non-small-cell lung cancer; ORR: objective response rate; OS: overall survival.

Twenty-one studies involving 4708 NSCLC patients were included in the association analysis between rs25487 (Arg399Gln) and ORR, of which 17, 17, 20, and 18 were for homozygous, heterozygous, dominant, and recessive models, respectively. There was high between-study heterogeneity in most of the comparisons (Table 2). Compared to the ArgArg genotype, GlnGln (OR = 1.71, 95% CI:1.16-2.52, p = .007, *I*^2^ = 56.8%, Figure 2(a)) or GlnArg (OR = 1.23, 95% CI:1.07-1.40, *p* = .003, *I*^2^ = 29.0%, Figure 2(b)) genotypes were associated with higher ORR. In the recessive model, GlnGln carriers had higher probability to respond to platinum-based chemotherapy (OR = 1.45, 95% CI:1.02-2.06, *p* = .037, *I*^2^ = 54.2%).

#### 3.2.2. Overall Survival and Progression-Free Survival

We included 12 and 4 studies in the meta-analysis regarding the association of rs25487 with OS or PFS, respectively. In the homozygous model, carriers of GlnGln had a favorable OS (HR = 0.60, 95% CI:0.40-0.88, *p* = .009, *I*^2^ = 63.2%, Figure 2(c)) and PFS (HR = 0.64, 95% CI:0.46-0.90, *p* = .010, *I*^2^ = 24.4%). However, no significant associations were found in the other comparisons (Table 2).

### 3.3. Rs1799782

#### 3.3.1. Objective Response Rate

**Figure 3:**
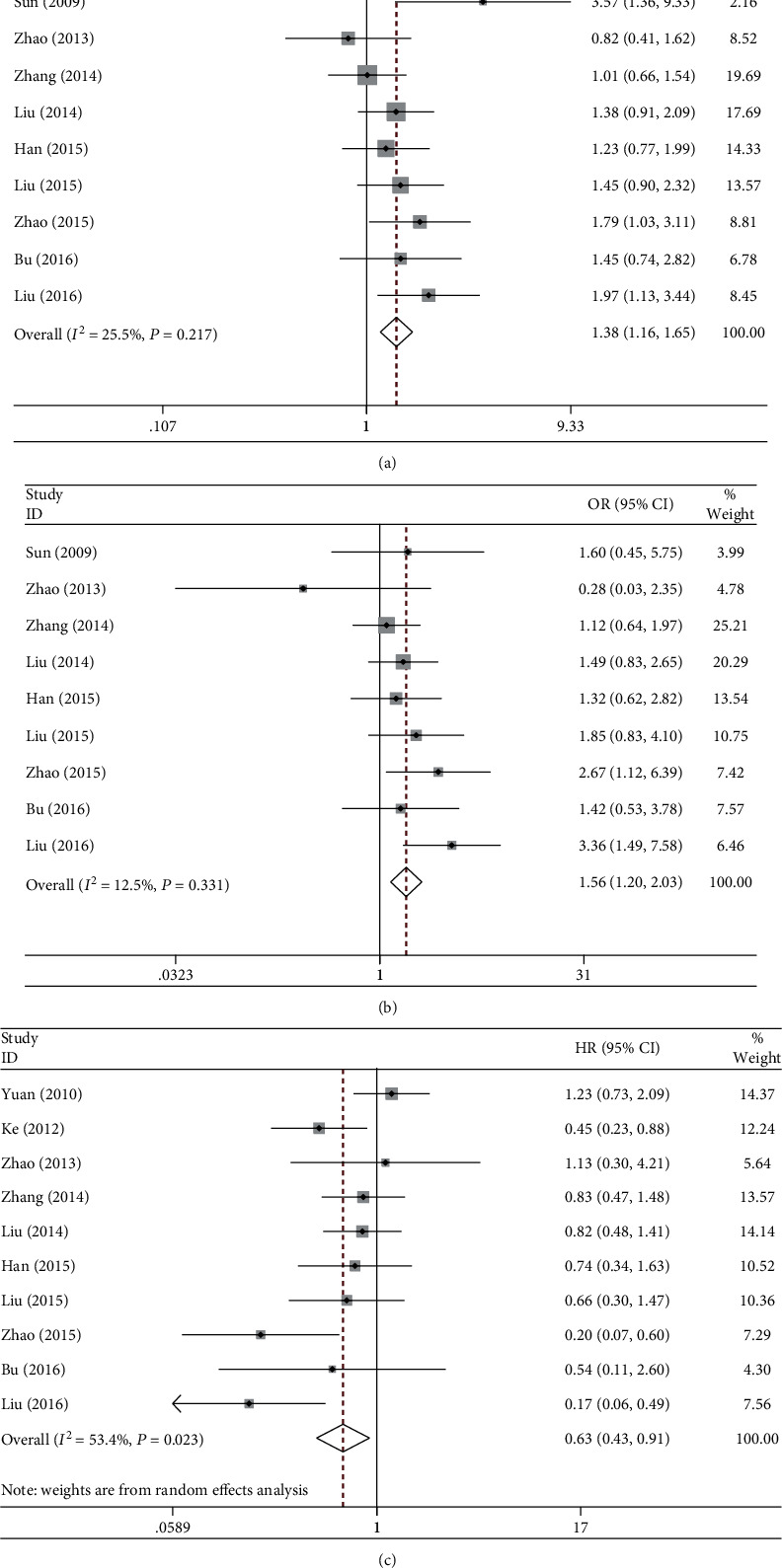
Forest plots of rs1799782 (Arg194Trp) and clinical outcomes in NSCLC patients under platinum-based chemotherapy. (a) ORR in the dominant model. (b) ORR in the recessive model. (c) OS in the homozygous model. NSCLC: non-small-cell lung cancer; ORR: objective response rate; OS: overall survival.

Nine studies, comprising 2228 NSCLC patients, with respect to the association between rs1799782 (Arg194Trp) and ORR of platinum-based chemotherapy, were included. Low or no heterogeneity was found, and fixed-effect model was used. After meta-analysis of all comparisons, Trp194 was found to be significantly associated with higher ORR (Table 2). Comparing to the homozygous wildtype, carrying at least one Trp allele was more likely to respond completely or partially to the chemotherapy (OR = 1.38, 95% CI:1.16-1.65, *p* < .001, *I*^2^ = 25.5%, Figure 3(a)). Significant associations were also found in homozygous, heterozygous, and recessive models Figure 3(b).

#### 3.3.2. Overall Survival and Progression-Free Survival

Compared to ArpArp, TrpTrp carriers had a favorable OS (HR = 0.63, 95% CI:0.43-0.91, *p* = .013, *I*^2^ = 53.4%, Figure 3(c)) after pooling 10 studies together, while TrpArg was not associated with OS (*p* = .173, *I*^2^ = 0). Only a few studies reported the association of rs1799782 with PFS, and none of the genotypes indicated a favorable PFS in pooling analysis (*p* > .05).

### 3.4. Sensitivity Analysis and Metaregression

Sensitivity analysis, by excluding one study each time and pooling the rest studies together, showed that none of a single study had significant impact on the pooled effect sizes of meta-analysis. Even after excluding two studies that comprised all stages of NSCLC patients 29^,^^33^, the effect sizes remained unchanged.

**Figure 4:**
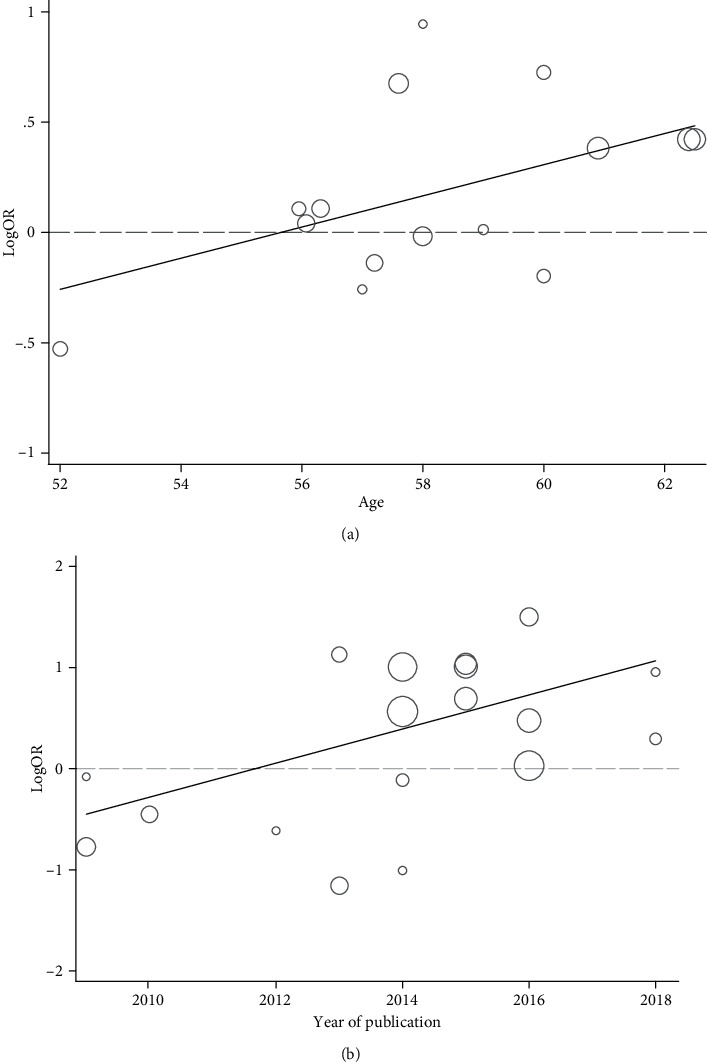
Meta-regression analysis with significant findings. (a) The effect of age on association of the rs25487 genotype with ORR in the heterozygous model. (b) The effect of year of publication on association of the rs25487 genotype with ORR in the recessive model. ORR: objective response rate.

For 25487, meta-regression analysis revealed an influence of age of participants in the heterozygous model (coefficient = 0.071, *p* = .029) and year of publication in the recessive model (coefficient = 0.18, *p* = .025), with ORR increasing with age and time (Figure 4). No similar associations were identified in other variables, particularly in the smoking rate (*p* > .05).

### 3.5. Publication Bias

The funnel plots in most of the meta-analyses were symmetric, and Egger's test indicated no evidence of publication bias (*p* > .10), except in the heterozygous model of 1799782 with ORR.

## 4. Discussion

*XRCC1* is the major component of the BER pathway, and its enhanced capacity to repair platinum-induced DNA damage may be correlated with the sensitivity to platinum drugs. Two mostly studied functional polymorphisms of *XRCC1*, rs25487 (Arg399Gln) and rs1799782 (Arg194Trp), are both located in the exons and modify the DNA repair activity. The Arg399Gln polymorphism occurs in the PARP-binding domain and may affect complex assembly or repair efficiency. The 399Gln allele was associated with elevated levels of aflatoxin B_1_-DNA adducts and glycophorin A somatic mutations^42^ and with a higher frequency of sister chromatid exchange^43^. Another polymorphism, Arg194Trp, resides in the linking region between the DNA polymerase *β* domain and PARP domain and was reported to disrupt the functionality of XRCC1 in hamsters^44^. Although the mechanisms have not been fully elucidated, the carriers of 399Gln and 194Trp are inferred to have deficient DNA repair activity and are more sensitive to platinum-based regimen. In accordance with this, several studies reported positive associations of rs25487 and rs1799782 with increased objective response rate and longer overall survival^27^^,^^29^^,^^45^. However, some other studies identified insignificant even negative associations^15^^,^^17^^,^^25^.

The present meta-analysis pooled all studies performed in Asian populations to further assessing the associations of both polymorphisms with platinum-based chemotherapy efficacy. For rs25487, carrying one or two 399Gln alleles had better ORR compared with the homozygous wildtype (OR = 1.23 and 1.71, respectively, *p* < .01). Meanwhile, the homozygous variant had longer overall survival and progression-free survival (HR = 0.60 and 0.64, respectively, *p* ≤ .010). However, obvious heterogeneity was found in most of the comparisons, and thus the results should be interpreted in caution. For rs1799782, significant correlations with ORR were identified in homozygous, heterozygous, dominant, and recessive models (all *p* values <.010), suggesting a gene dosage effect of positive association. Additionally, the homozygous 194Trp was associated with a favorable OS (HR = 0.63, *p* = .013).

The present study mainly focused on the Asian populations. Several studies have reported controversial associations with ORR, OS, or PFS in the Caucasians^15^^–^17^,^^45^^–^49. We found, in subanalysis of Caucasians, significant associations of rs25487 with a shorter OS in the homozygous model (HR = 2.72, *p* = .001, *I*^2^ = 49.4%) by pooling 3 studies and with a decreased ORR in the dominant model (OR = 0.68, *p* = .031, *I*^2^ = 0) by pooling 5 studies. However, there seems a contradictory role of rs25487 in clinical outcomes between different ethnicities since it predicted longer OS and increased ORR in Asians. This may be caused by various factors, such as genetic background, sample size, and dietary or living habits. This discrepancy needs further confirmation by including more investigations with larger sample size in Caucasians. Since only a few studies in Caucasians were available, we gave up the subanalysis of Caucasians in the present study. Compared with previous meta-analyses^50^^–^52, we included more eligible studies and a larger sample size of more than 5500 patients under similar genetic background, indicating a stronger robustness of our results.

Our results revealed that two functional polymorphisms of *XRCC1* may have prognostic value of NSCLC patients under platinum-based chemotherapy, but they are still far away from clinical practice, since many genes in other pathways, apart from the BER pathway, also contribute to the sensitivity to platinum drugs 53. The NER pathway, through *ERCC1*^47^^,^^54^, *ERCC2*^55^, and *ERCC5*^33^, also played a pivotal role in repairing DNA lesions and was reported to be associated with clinical outcomes. The other genes, including *XRCC3*^46^ in the DSB pathway, *TP53*^56^ and *MDM2*^57^ in the p53 pathway, *AKT1*^58^ in the PI3K/PTEN/AKT pathway, *SMAD3*^59^ in the TGF-*β* pathway, and *MTHFR*^60^ involved in folate metabolism, were also found in association with ORR, OS, or PFS in NSCLC patients using platinum-based regimen. The combinations of various markers should be furtherly investigated for the prognostic value in NSCLC patients under platinum-based chemotherapy.

There are several limitations in our mate-analysis. First, an ideal meta-analysis should be based on individual-level data, especially in the analysis of OS and PFS, although it is usually quite difficult to obtain these data. The interpretation of our results in terms of OS and PFS should be cautious. Secondly, we did not analyze the interaction of both polymorphisms in predicting the clinical outcomes because of a lack of sufficient data. Thirdly, there were substantial between-study heterogeneities in the pooling analysis of rs25487, and thus more studies are needed in the future.

## 5. Conclusion

In conclusion, our meta-analysis suggested that two functional polymorphisms of *XRCC1*, rs25487 and rs1799782, can be used as prognostic factors for platinum-based chemotherapy in Asian patients with NSCLC.

## Figures and Tables

**Table 1:** Basic characteristics of all included studies.

Author	Year	Country	Tumor stage	Sample size (male/female)	Median age (year)	Smoking rate (%)	Genotyping method	SNPs	Clinical outcomes	NOS
Sun	2009	China	IV	82 (53/29)	59	NR	DNA microarray	rs25487, rs1799782	ORR	6
Yao	2009	China	IIIB-IV	108 (71/38)	61	NR	PCR-RFLP	rs25487	ORR, OS	6
Yuan	2010	China	III-IV	199 (129/70)	56	59.3	PCR-RFLP	rs25487, rs1799782	OS, PFS	7
Shiraishi	2010	Japan	III-IV	201 (136/65)	57.2	63.2	TaqMan	rs25487	ORR	6
Li	2011	China	III-IV	89 (64/25)	59.1	NR	PCR-RFLP	rs25487	ORR	7
Zhou	2011	China	IV	111 (67/44)	57	NR	Direct sequencing	rs25487	ORR	7
Liao	2012	China	IIIB-IV	62 (35/27)	57	NR	SNPstream	rs25487	ORR, OS	6
Ke	2012	China	I-IV	460 (334/126)	59.5	67.4	PCR-CTTP	rs25487, rs1799782	OS	6
Zhao	2013	China	IIIB-IV	147 (92/55)	60	45.9	TaqMan	rs25487, rs1799782	ORR, OS, PFS	7
Lee	2013	South Korea	III-IV	382 (311/71)	NR	83.2	MALDI-TOF	rs25487	ORR, OS	6
Zhang	2014	China	III-IV	375 (249/126)	60.9	NR	MALDI-TOF	rs25487, rs1799782	ORR, OS, PFS	6
Peng	2014	China	III-IV	235 (180/55)	58	61.3	PCR-CTTP	rs25487	ORR	6
Liu	2014	China	I-IV	378 (297/81)	62.4	56.1	MALDI-TOF	rs25487, rs1799782	ORR, OS, PFS	7
Du	2014	China	III-IV	161 (108/53)	60	54.7	qPCR	rs25487	ORR	7
Deng	2015	China	IIIB-IV	97 (66/31)	57	40.2	Direct sequencing	rs25487	ORR, PFS	6
Han	2015	China	IIIB-IV	325 (116/209)	57.6	68.3	PCR-RFLP	rs25487, rs1799782	ORR, OS	7
Liu	2015	China	IIIB-IV	322 (226/96)	62.5	43.5	PCR-RFLP	rs25487, rs1799782	ORR, OS	6
Zhao	2015	China	III-IV	206 (124/82)	56.1	65.5	PCR-RFLP	rs25487, rs1799782	ORR, OS	7
Chen	2016	China	III-IV	1024 (724/300)	NR	55.6	MALDI-TOF	rs25487	ORR	6
Bu	2016	China	III-IV	141 (100/41)	55.9	66.0	PCR-RFLP	rs25487, rs1799782	ORR, OS	6
Liu	2016	China	IIIB-IV	252 (104/148)	56.3	75.4	PCR-RFLP	rs25487, rs1799782	ORR, OS	7
Liao	2018	China	IV	58 (39/19)	58	44.8	TaqMan	rs25487	ORR, OS	7
Dong	2018	China	IIIB-IV	152 (101/51)	52	52.0	MALDI-TOF	rs25487	ORR	6

PCR-RFLP: polymerase chain reaction restriction fragment length polymorphism; PCR-CTTP: PCR with the confronting-two-pair primer; MALDI-TOF: matrix-assisted laser desorption/ionization time-of-flight mass spectrometry; ORR: objective response rate; OS: overall survival; PFS: progression-free survival; NR: not reported. NOS: Newcastle-Ottawa Scale.

**Table 2:** Meta-analysis of association between rs25487 or rs1799782 and efficacy of NSCLC treated with platinum-based chemotherapy in Asians.

XRCC1	ORR				OS				PFS			
	No.	*I* ^2^ (%)	OR (95% CI)	*p*	No.	*I* ^2^ (%)	HR (95% CI)	*p*	No.	*I* ^2^ (%)	HR (95% CI)	*p*
rs25487 (Arg399Gln)^$^												
Homozygous model	17	56.8	1.71 (1.16-2.52)	.007	12	63.2	0.60 (0.40-0.88)	.009	4	24.4	0.64 (0.46-0.90)	.010
Heterozygous model	17	29.0	1.23 (1.07-1.40)	.003	12	63.2	0.81 (0.64-1.03)	.083	4	0	0.87 (0.71-1.07)	.193
Dominant model	20	69.3	1.11 (0.87-1.41)	.386	5	39.4	1.00 (0.79-1.27)	.980	4	0	0.87 (0.71-1.08)	.215
Recessive model	18	54.2	1.45 (1.02-2.06)	.037	3	13.8	1.14 (0.82-1.59)	.490	—	—	—	—
rs1799782 (Arg194Trp)^#^												
Homozygous model	9	34.0	1.73 (1.31-2.27)	<.001	10	53.4	0.63 (0.43-0.91)	.013	4	0	0.93 (0.68-1.26)	.627
Heterozygous model	9	0	1.28 (1.06-1.55)	.007	10	0	0.89 (0.70-1.05)	.173	4	0	1.02 (0.83-1.26)	.838
Dominant model	9	25.5	1.38 (1.16-1.65)	<.001	4	0	0.91 (0.71-1.16)	.446	3	0	1.05 (0.83-1.34)	.662
Recessive model	9	12.5	1.56 (1.20-2.03)	<.001	—	—	—	—	—	—	—	—

^$^Homozygous, heterozygous, dominant, and recessive models indicated GlnGln vs. ArgArg, GlnArg vs. ArgArg, GlnGln+GlnArg vs. ArgArg ,and GlnGln vs. GlnArg+ArgArg, respectively. ^#^Homozygous, heterozygous, dominant, and recessive models indicated TrpTrp vs. ArgArg, TrpArg vs. ArgArg, TrpTrp+TrpArg vs. ArgArg, and TrpTrp vs. TrpArg+ArgArg, respectively. Abbreviations: NSCLC: non-small-cell lung cancer; ORR: objective response rate; OS: overall survival; PFS: progression-free survival; OR: odds ratio; HR: hazard ratio.
